# Effective composite grafting of sub coronal penile amputation after ritual circumcision(khatna) in a 3-year-old child

**DOI:** 10.1016/j.eucr.2026.103397

**Published:** 2026-03-05

**Authors:** Brijesh Mishra, Bh Gowtham reddy, Ravi kumar

**Affiliations:** Department of Plastic and Reconstructive Surgery, King George's Medical University, Lucknow, India

## Abstract

Ritual Circumcision is common practice for male children, in Indian Muslims, but if it is done by unsafe local barbers may result in partial or total penile amputation. We report a case of 3-year-old boy presented with total sub coronal penile amputation during ritual circumcision. An attempt at replantation was unsuccessful due to the small lumen of the corporal arteries (<0.1mm) despite a successful dorsal venous anastomosis. A composite graft of the amputated glans was used with dorsal venous anastomosis was utilized with successful take of the composite graft.

## 1 Introduction

The term Circumcision is derived from Latin word circumcidere, meaning "to cut around".^1^ Ritual Circumcision (khitan or khatna) is common practice in male children, especially in Jewish and Muslim cultures, when done by medical professionals it has good health benefits such as protection against penile cancer, recurrent urinary tract infection, balanitis and sexually transmitted diseases 2, 3, 4.But if circumcision is performed by untrained or poorly trained individuals it may result in complications ranging from minor (bleeding, infection, scarring, meatal stenosis) to major (partial or total penile amputation) complications. We are reporting here a case of total penile amputation at sub coronal level during ritual circumcision in a 3-year-old boy by a local barber at home.

## 2 Case report

**Fig. 1:**
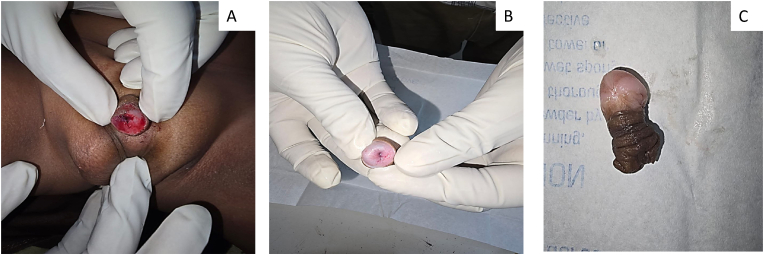
Subcoronal Penile Amputation at Presentation and Immediate Operative Reconstruction (A) Amputated penile stump at sub coronal level. (B) Amputated penile part inside the avulsed skin flap. (C) Amputated penile part including glans and avulsed skin flap.

**Fig. 2:**
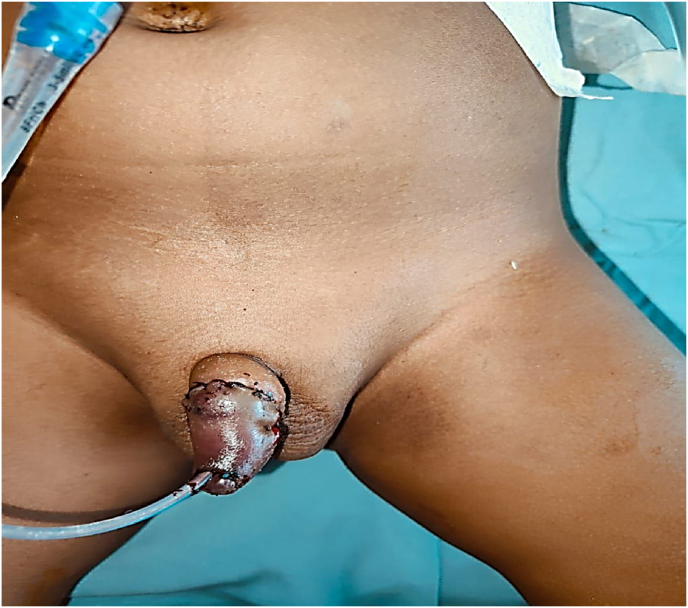
Immediate postoperative view following composite grafting showing good composite graft alignment, interrupted skin sutures, urinary catheter in situ.

**Fig. 3:**
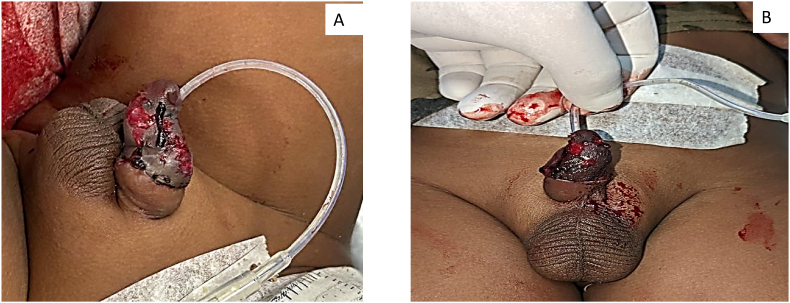
Postoperative day 7 of distal penile composite graft showing venous congestion followed by intra graft heparin injection. (A) Dorsal view (B) Ventral view.

**Fig. 4:**
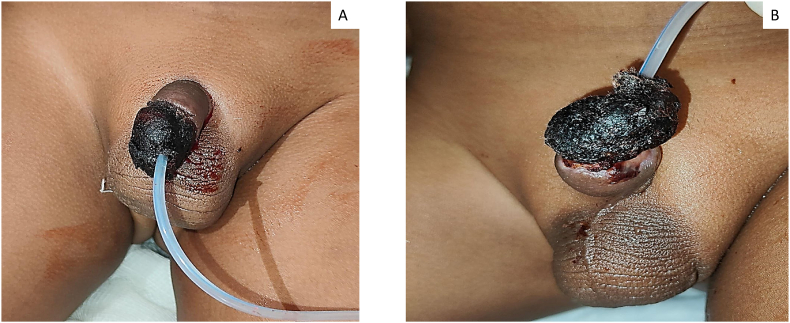
Postoperative Day 13, showing black eschar formation of overlying skin flap of the composite graft with urinary catheter in situ.(A) Dorsal view (B) Lateral view.

**Fig. 5:**
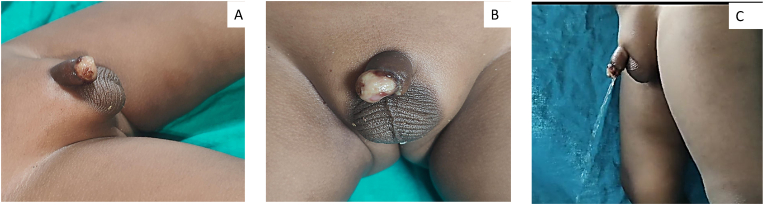
Postoperative Day 20, showing healthy glans indicating composite graft take with loss of avulsed skin flap (A) Lateral view (B) Dorsal view demonstrating satisfactory composite graft take. (C) Clinical image showing normal urinary stream through the reconstructed penile segment, indicating adequate urethral patency and functional success of the composite graft.

Parents of a 3-year-old boy has conducted religious ritual circumcision ceremony(sunnah) on February 12, 2025 at around 11 a.m. at Sitapur, during which a local barber has performed circumcision at home by unsafe method pulling the prepuce forward and excising with a sharp knife with resultant total penile amputation at the sub coronal level. Patient was immediately taken to district hospital, Sitapur where primary management was done and later patient was referred to king George's medical university, Lucknow for further management with amputated part in ice bag on same day at 3:37 p.m. (Fig. 1A,B,1C). After initial resuscitation and blood workup patient was shifted to Plastic and reconstructive surgery department operation theatre around 7:45 p.m. Initially microsurgical dissection of corporal arteries and dorsal penile artery, vein and nerve were done, but only dorsal venous anastomosis with composite grafting was done because of very small vessel lumen size (<0.1mm) of corporal arteries, arterial anastomosis was not possible in this case because of micro instruments in our setup was not negotiable for small lumen size of corporal arteries,So we have proceeded for composite graft after inserting 8F silicone foley's catheter through amputated part and residual penile stump, where first corpus spongiosum was repaired over the 8F floey's stent in the urethra using 7-0 nylon followed by approximation of corpora cavernosa using 6-0 vicryl suture followed by closure of buck's facia and dorsal venous anastomosis was done by 10 -0 nylon, followed by closure of skin with 6-0 nylon(Fig. 2). Initially postoperative period venous congestion was noted over composite graft of amputated part, we had given chemical leech therapy by injecting diluted heparin intra replant from post operative day 1 to day 10 (Fig. 3A,B), later skin over the penile amputated part became black on postoperative day 13(Fig. 4A,B). We have done debridement of blackish discoloured skin, inside the skin flap good take of glans tissue was noted. Foley's catheter was removed on post op day 20, normal single urine stream from the external urethral meatus present (Fig. 5A,B,5C).

## 3 Discussion

Male circumcision is a major part of the ritual (sunnah) in the religion of Islam and therefore is common practice among Muslims in India. Nearly 25%–30% of the world's male population are circumcised 5, 6, 7, 8. Studies have shown that neonatal circumcision is associated with the least risks,^9^ and the highest medical benefits and complication rates increase with the increasing age.^10^ When procedure performed by trained professionals, early male circumcision in the neonatal period is easy, less time-consuming, with fast healing and low rates of minor adverse events (0.2%–0.4%).^11^ The Complications after neonatal circumcision were observed by Sebastian O.Ekenze et al. in their study and reported in 2013. They found glanular adhesion, meatal stenosis, urethrocutaneous fistula, trapped penis, implantation dermoid, and glans amputation after circumcision.^12^

Penile replantation was first reported by Ehrich WS (1929) in self-inflicted penile amputation by a man with radial saw.^13^ We have done composite grafting of penile amputation at sub coronal level with good take of composite graft and effective results. Similar replantation was done by Bouassida Khaireddine et al. (2012) in two cases and found good results.^14^

## 4 Conclusion

Ritual Circumcision(khatna) is a common practice in Islam followed worldwide. Reasons behind circumcision are different, but it should be performed by experienced medical practitioner.

If microvascular anastomosis is not possible due to small vessel lumen (less than 0.1mm) at sub coronal level then composite grafting of amputated penile part will give good and effective results.

## CRediT authorship contribution statement

**Brijesh Mishra:** Validation, Supervision, Project administration, Conceptualization. **Bh Gowtham reddy:** Writing – review & editing, Writing – original draft, Visualization, Resources, Methodology, Investigation, Formal analysis, Data curation. **Ravi kumar:** Supervision, Methodology.

## Financial disclosure

No financial conflicts of interest.
